# Supplementary material for: Detailed analysis of distorted retinal and its interaction with surrounding residues in the K intermediate of bacteriorhodopsin
Source: Commun Biol. 2023 Feb 17;6:190. doi: 10.1038/s42003-023-04554-2 (PMC9938236; doi:10.1038/s42003-023-04554-2)
Supplement: Supplementary file 2 — Supplementary Information [file 42003_2023_4554_MOESM2_ESM.pdf]

Supplementary information

Detailed analysis of distorted retinal and its interaction with surrounding residues in the K intermediate of bacteriorhodopsin

Shoun Taguchi[1], Satomi Niwa[1], Hoang-Anh Dao[1], Yoshihiro Tanaka[1], Ryota Takeda[1], Shuya Fukai[1], Kazuya Hasegawa[2] and Kazuki Takeda[1*]

[1]Department of Chemistry, Graduate School of Science, Kyoto University, Sakyo-ku, Kyoto 606-8502, Japan

[2]Structural Biology Division, Japan Synchrotron Radiation Research Institute (JASRI), 1-1-1 Kouto, Sayo-cho, Sayo-gun, Hyogo 679-5198, Japan

[*]Correspondence email: ktakeda@kuchem.kyoto-u.ac.jp

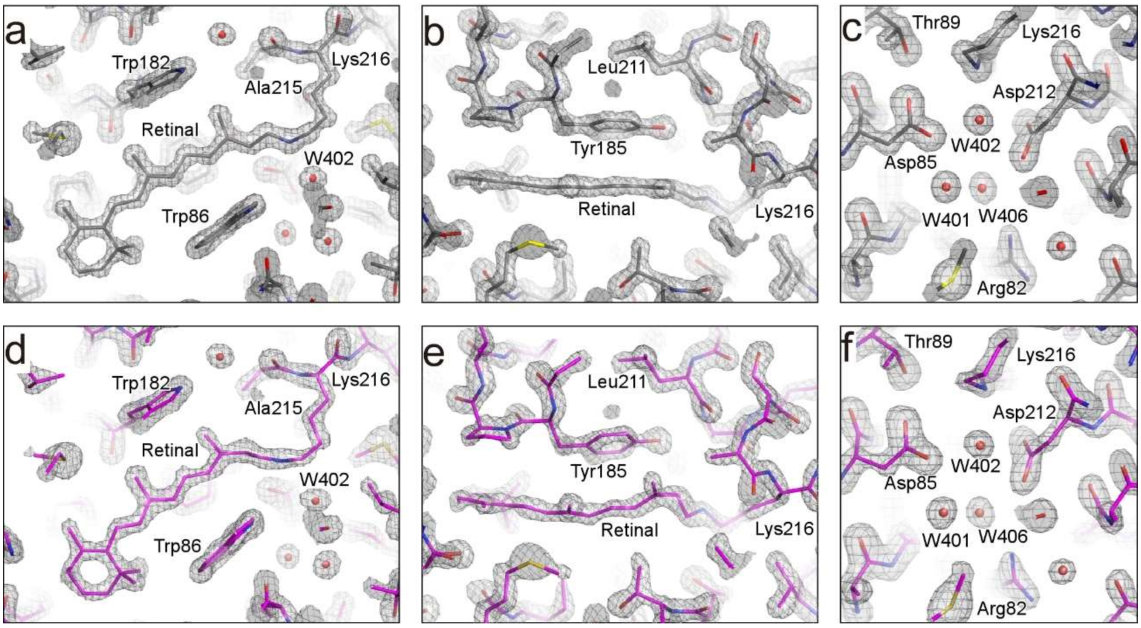

Supplementary Figure 1. The 2F_{obs} – F_{calc} electron density maps around retinal. a. The map for the ground state is shown at a contour level of 2.5σ. **b.** The top view of a. **c.** A view from the right side of a. **d.** The map for the K intermediate. **e.** The top view of d. **f.** A view from the right side of d.

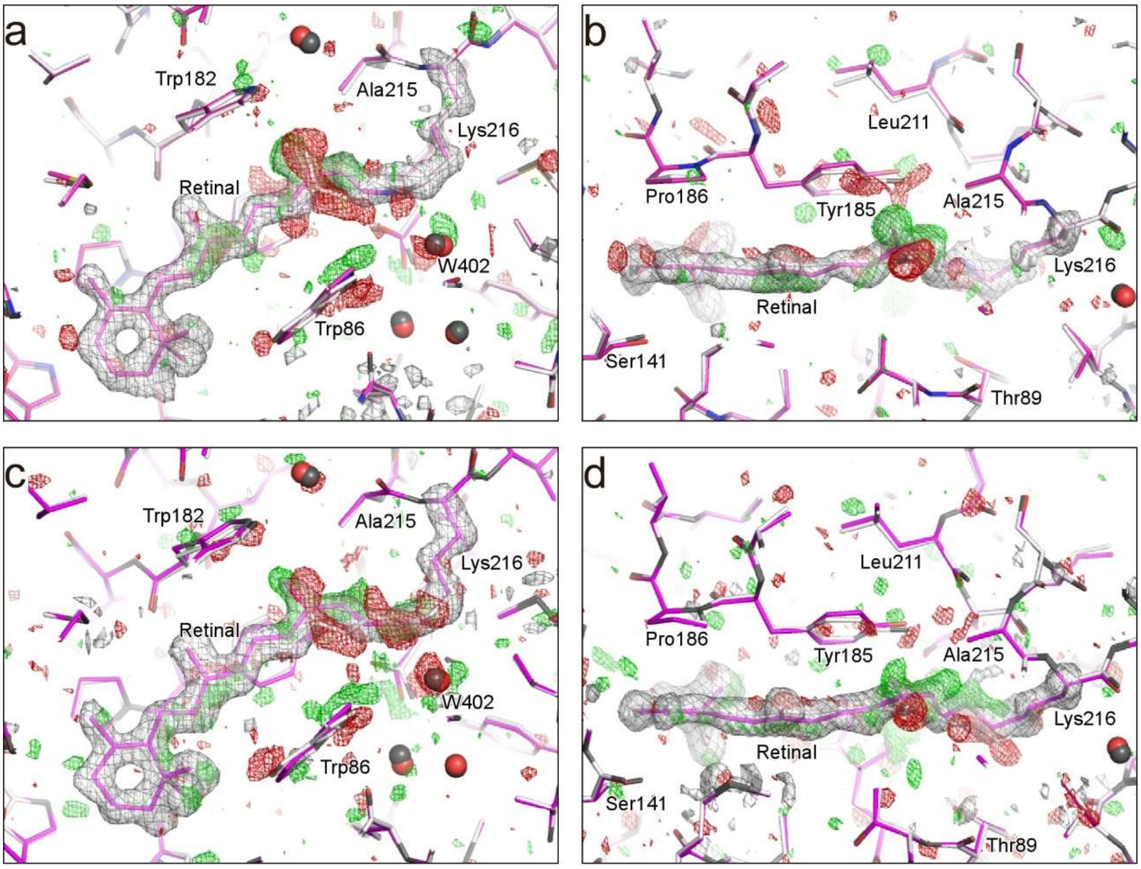

Supplementary Figure 2. The *F*(K+bR) – *F*(bR) difference map around retinal of additional data. a. The map for crystal II is shown as green and red meshes at contour levels of ±3σ. The omit map for retinal and the Lys216 side chain calculated from the extrapolated data is overlaid as gray meshes at a contour level of +3σ. **b.** The top view of a. **c.** The map for crystal III. **d.** The top view of c.

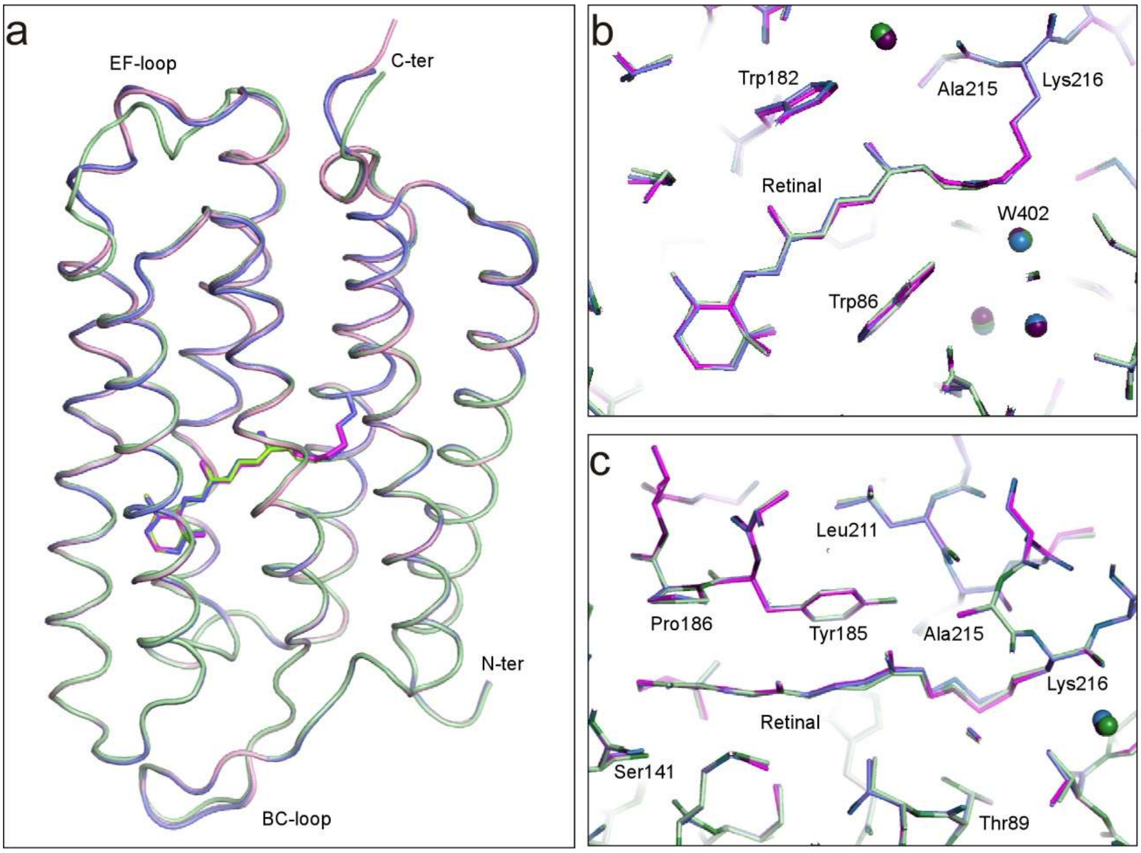

Supplementary Figure 3. Superimposition of three K structures.
a. The whole view is shown as a tube model while retinal is shown as sticks. K structures from crystal I, crystal II and crystal III are colored in magenta, green and blue, respectively. **b.** A close-up view of the superimposition around retinal. **c.** A side view of b.

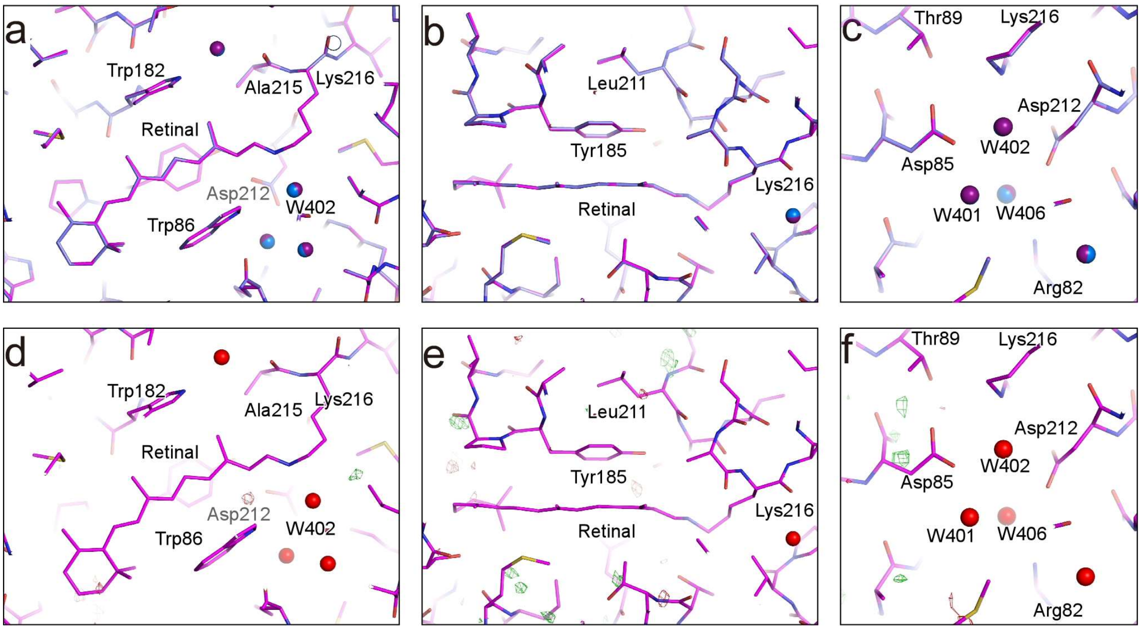

Supplementary Figure 4. Structural comparison of structures in the ground state. **a.** Carbon atoms in the ground state structures from crystal I and crystal III are colored in magenta and blue, respectively. **b.** The top view of a. **c.** A view from the right side of a. **d.** The F_{I}(bR) – F_{III}(bR) difference map around retinal is shown as green and red meshes at contour levels of ±3σ. The structure factors from a single component of the twinned crystals were derived by the method of Pratt *et al.*,[52]. **e.** The top view of d. **f.** A view from the right side of d.

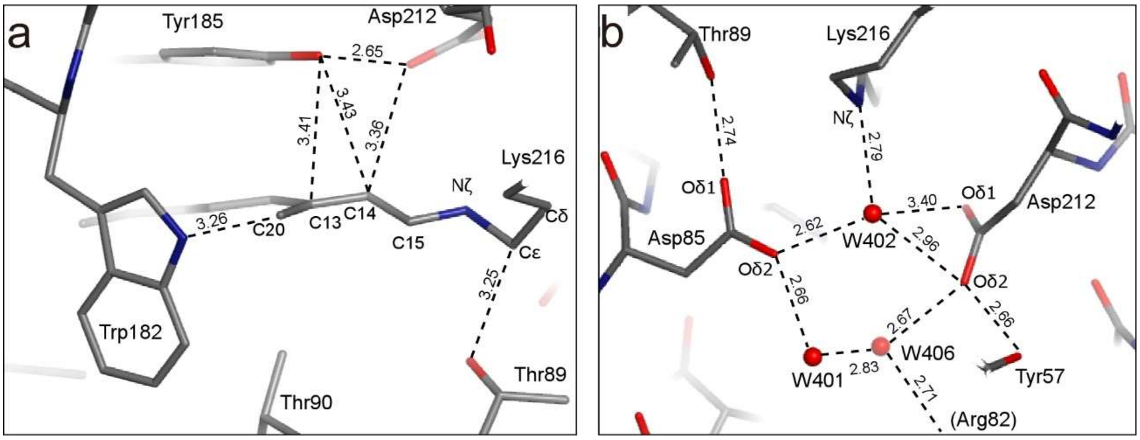

Supplementary Figure 5. Structure of the ground state. a. Interatomic distances of less than 3.5 Å. **b.** Hydrogen bonds with distances of less than 3.5 Å.

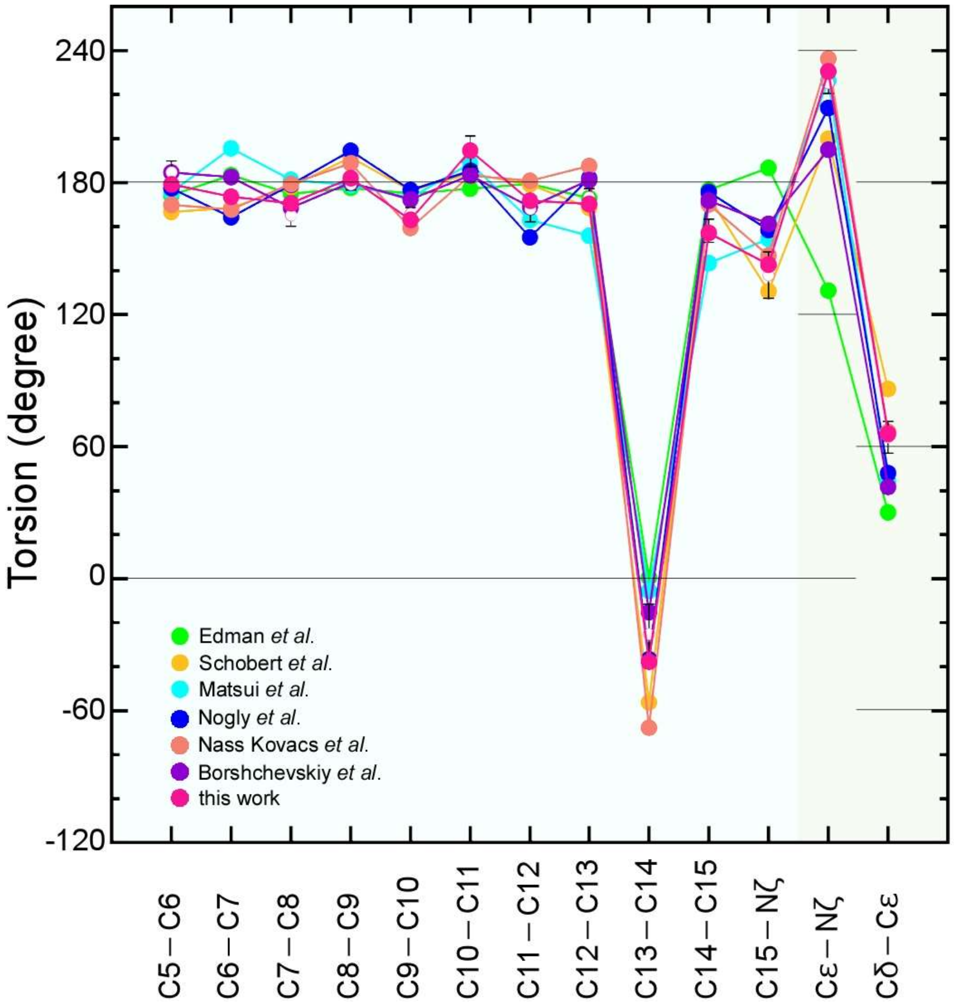

Supplementary Figure 6. Comparison of the torsion angles of various K structures. Torsion angles along the retinal polyene chain in the K structure are plotted in green (1QK0 by Edman *et al.*)[13], yellow (1M0K by Schobert *et al.*)[14], cyan (1IXF by Matsui *et al.*)[15], navy (6G7K by Nogly *et al.*)[18], orange (6GA6 by Nass Kovacs *et al.*)[19], purple (7Z0C by Borshchevskiy *et al.*)[16] and magenta (7XJC in this work). Hollow circles and error bars are the mean values and the standard deviations calculated from three structures of this work.

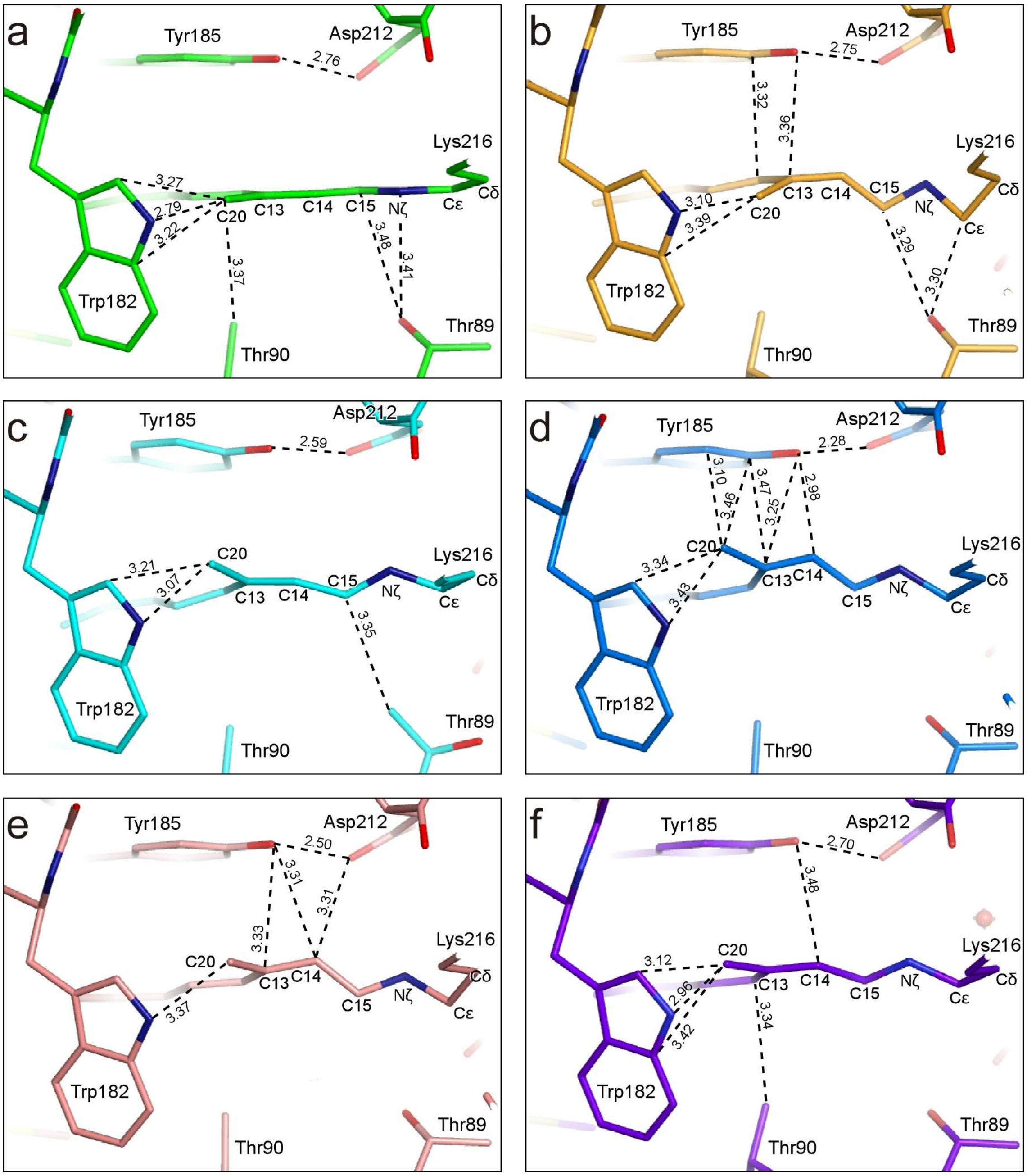

Supplementary Figure 7. Comparison of interactions between retinal and the surrounding residues. a. Interatomic distances for the K structure in 1QK0[13]. Interactions with distances of less than 3.5 Å are shown. **b.** The structure in 1M0K[14]. **c.** The structure in 1IXF[15]. **d.** The structure in 6G7K[18]. **e.** The structure in 6GA6[19]. **f.** The structure in 7Z0C[16].

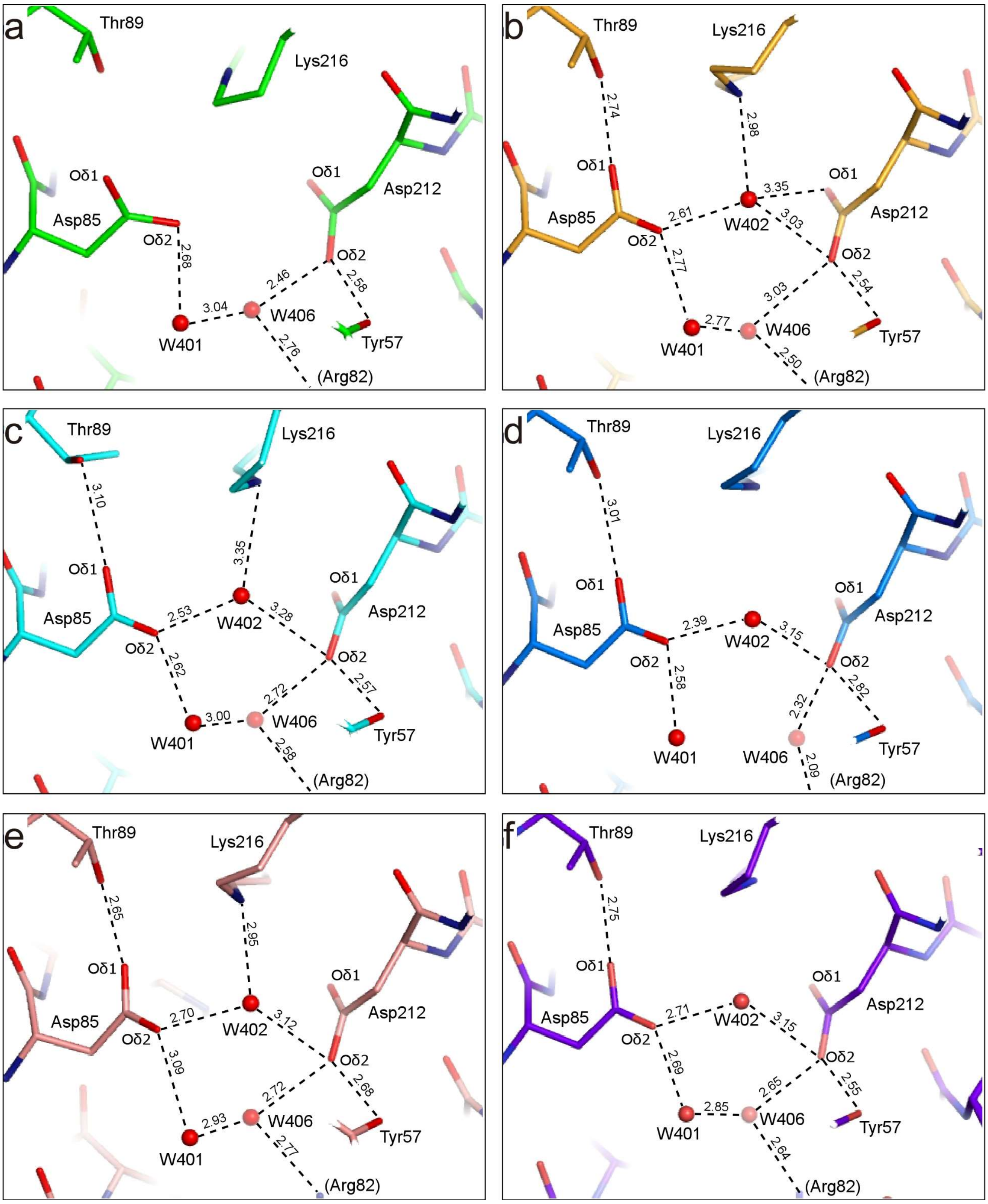

Supplementary Figure 8. Comparison of hydrogen bonding in the EC side of the SB linkage. a. Hydrogen bonding distances in 1QK0[13]. Hydrogen bonds with distances of less than 3.5 Å are shown. **b.** The distances in 1M0K[14]. **c.** The distances in 1IXF[15]. **d.** The distances in 6G7K[18]. **e.** The distances in 6GA6[19]. **f.** The distances in 7Z0C[16].

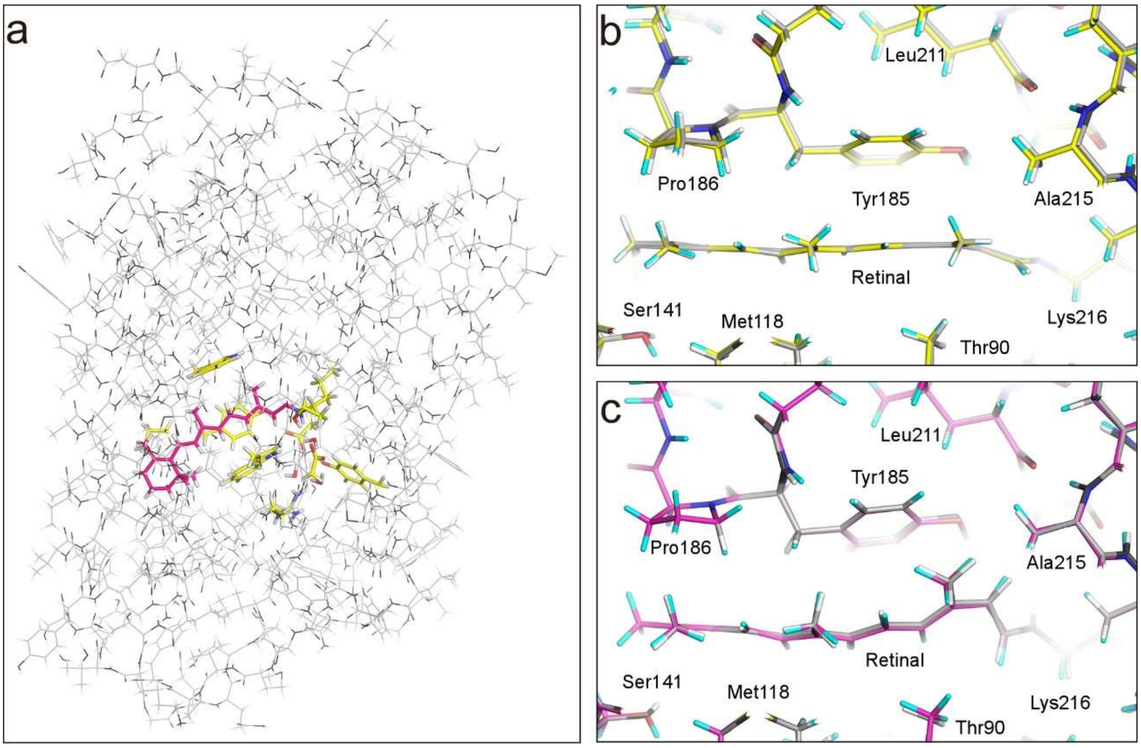

Supplementary Figure 9. The QM/MM calculation. a. A whole view. Atoms indicated as sticks are in the QM region, while the atoms indicated as gray lines are in the MM region. The QM region includes Tyr57, Arg82, Asp85, Trp86, Thr89, Trp182, Tyr185, Pro186, Asp212, Lys216, retinal, W401, W402 and W406. **b.** The optimized structure of the ground state. The gray sticks are for the model in which only coordinates of hydrogen atoms are optimized, while the colored sticks are for the model in which coordinates of all atoms are optimized. **c.** The optimized structure of the K intermediate.

Supplementary Table 1. Summary of reported K structures.

PDB-ID	1QK0	1M0K	1IXF	6G7K	6GA6	7Z0C	7XJC
Space group	$P6_3$	$P6_3$	$P622$	$P6_3$	$P6_3$	$P6_3$	$P6_3$
Width and thickness of hexagonal plate crystal (µm)	70, 5−10	80, 10−15	200, 50	15−50, 1−5	20−30, 2−3	250−600, 10−70	350, 30
Method	Cryo trapping	Cryo trapping	Cryo trapping	TR-SFX (10 psec)	TR-SFX (10 psec)	Cryo trapping	Cryo trapping
Laser wavelength (Å)	532	532	532	529	520	530	532
Laser power (mW or µJ/pulse)	100 mW	5 mW	−	17 µJ/pulse	5.9 µJ/pulse	0.3 mW	1 mW
Laser power density (W/cm^2)	30	−	0.1	1×10^{12}	0.5×10^{12}	−	0.1
Temperature at K generation (K)	110	100	100	RT	RT	100	100
Temperature at measurement (K)	110	100	100	RT	RT	100	15
X-ray source[a]	SR	SR	SR	X-FEL	X-FEL	SR	SR
X-ray damage	not cared	not cared	low	free	free	low	low
Dose (MGy)	−	−	0.1	−	−	0.03 or 0.1	0.05
Resolution (Å)	2.1	1.43	2.6	1.9	1.8	1.53	1.33
Twin ratio	<0.02	0.49	0.00	0.00	0.074	0.00	0.20
Fraction of K	0.35	0.40	0.40	0.16	0.10	0.15	0.19
Distance of Nζ−W402 (Å)	−	2.98	3.35	4.07	2.95	3.57	3.07
Reference	13	14	15	18	19	16	this work

[a] SR: synchrotron radiation, X-FEL: X-ray free electron laser

Supplementary Table 2. Data collection and refinement statistics of Crystal II and III.

Data	bR + K	bR	K_ext	bR + K	bR	K_ext
**Data collection**						
Crystal	II	II	II	III	III	III
Cell dimensions, *a*, *c* (Å)	60.44, 107.32	60.44, 107.32	60.44, 107.32	60.62, 110.64	60.63, 110.63	60.62, 110.64
Resolution (Å)	50−1.34 (1.42–1.34)[a]	50−1.34 (1.42–1.34)	−	50−1.36 (1.44–1.36)	50−1.36 (1.44–1.36)	−
R_{sym}[b](%)	8.7 (113.3)	8.7 (113.9)	−	8.2 (105.2)	8.3 (106.2)	−
$I/\sigma(I)$	10.1 (1.2)	9.8 (1.2)	−	10.2 (1.3)	10.1 (1.3)	−
Completeness (%)	99.9 (99.8)	99.9 (99.9)	−	99.7 (99.1)	99.8 (99.2)	−
Redundancy	5.8 (5.5)	5.8 (5.5)	−	5.7 (5.5)	5.7 (5.5)	−
$CC_{1/2}$ (%)	99.8 (43.6)	99.8 (41.2)	−	99.8 (46.1)	99.8 (47.0)	−
**Refinement**						
Resolution (Å)	50−1.34	50−1.34	20−1.34	50−1.36	50−1.36	20−1.36
Twin ratio	0.42	0.42	0.42	0.22	0.22	0.17
Fraction of K	0.34	0.0	1.0	0.21	0.0	1.0
R_{work}[c]/ R_{free}[d] (%)	14.4/17.4	12.7/17.6	22.4/25.7	17.0/18.0	16.2/17.8	28.1/28.6

[a]Values for the highest resolution shell are in parentheses.

[b]$R_{sym} = \Sigma_{hkl}\Sigma_i| I_{hkl,I} - \langle I_{hkl}\rangle|/\Sigma_{hkl}\Sigma_i I_{hkl,i}$.

[c]$R_{work} = \Sigma_{hkl}||F_{obs}| - |F_{calc}||/\Sigma_{hkl}|F_{obs}|$.

[d]R_{free} was calculated by using 5% of the reflections that were not included in the refinement as a test set.

Supplementary Table 3. Hydrogen bonding distances around retinal.

State			K		Ground state		
Hydrogen bonding pair			d_{K} (Å)[a]	s_{K} (Å)[b]	d_{bR} (Å)[a]	s_{bR} (Å)[b]	σ level[c]
Lys216 Nζ	–	Asp212 Oδ1	3.27	0.10	3.93	0.06	6.5
Lys216 Nζ	–	W402	3.07	0.04	2.79	0.02	6.3
Thr89 Oγ1	–	Asp85 Oδ1	2.71	0.04	2.74	0.01	0.7
W402	–	Asp85 Oδ2	2.75	0.12	2.62	0.05	1.0
W402	–	Asp212 Oδ1	3.08	0.15	3.40	0.05	2.1
W402	–	Asp212 Oδ2	2.96	0.20	2.96	0.05	0.0
W401	–	Asp85 Oδ2	2.71	0.14	2.66	0.04	0.3
W401	–	W406	2.72	0.05	2.83	0.04	1.7
W406	–	Asp212 Oδ2	2.76	0.15	2.67	0.02	0.6
Arg82 Nη	–	W406	2.53	0.06	2.71	0.02	2.8

[a] Distances (d_{K} and d_{bR}) are for the structures of Crystal I.

[b] Standard deviations (s_{K} and s_{bR}) were calculated from structures of three crystals.

[c] σ level = $|d_{K} - d_{bR}| / (s_{K}^{2} + s_{bR}^{2})^{1/2}$.

Supplementary Table 4. Torsion angle for the polyene chain of retinal in K.

Bonds	Crystal I	Crystal II	Crystal III	Mean	s.d.[a]
C5–C6	179	-175	-171	-176	5
C6–C7	174	177	171	174	3
C7–C8	171	164	161	165	5
C8–C9	-178	178	178	179	2
C9–C10	163	166	169	166	3
C10–C11	-165	-173	-159	-166	7
C11–C12	172	161	171	168	6
C12–C13	170	178	172	173	4
C13–C14	-38	-11	-27	-25	14
C14–C15	157	153	164	158	5
C15–Nζ	143	126	145	138	11
Nζ–Cε	-129	-137	-138	-135	5
Cε–Cδ	66	71	56	64	7

[a] Standard deviations were calculated from torsions of the three crystals.
