# Supplementary material for: Detailed analysis of distorted retinal and its interaction with surrounding residues in the K intermediate of bacteriorhodopsin
Source: Commun Biol. 2023 Feb 17;6:190. doi: 10.1038/s42003-023-04554-2 (PMC9938236; doi:10.1038/s42003-023-04554-2)
Supplement: Supplementary file 3 — Description of Additional Supplementary Files [file 42003_2023_4554_MOESM3_ESM.docx]

**Description of Additional Supplementary Files**

**File name:** Supplementary Data

**Description:** The source data for Figures 2b and 5b in the paper
